# Blood-Derived Eye Drops for the Treatment of Corneal Neuropathic Pain

**DOI:** 10.1089/jop.2023.0155

**Published:** 2024-06-17

**Authors:** Ansa Anam, Chang Liu, Louis Tong, Yu-Chi Liu

**Affiliations:** ^1^ Department of Ophthalmology, MTI Khyber Teaching Hospital, Peshawar, Pakistan.; ^2^ Cornea and Refractive Surgery Group, Singapore Eye Research Institute, Singapore, Singapore.; ^3^ Department of Corneal and External Eye Disease, Singapore National Eye Centre, Singapore, Singapore.; ^4^ Ophthalmology and Visual Sciences Academic Clinical Program, Duke-NUS Medical School, Singapore, Singapore.; ^5^ Ocular Surface Group, Singapore Eye Research Institute, Singapore, Singapore.

**Keywords:** blood-derived preparations, neuropathic corneal pain, ocular surface conditions, inflammation, growth factors

## Abstract

Blood-derived preparations, including autologous or allogenic serum, umbilical cord serum/plasma, and platelet-rich plasma eye drops, contain various growth factors, cytokines, and immunoglobulins that resemble natural tears. These components play important roles in corneal cell migration, proliferation, and wound healing. Blood-derived eye drops have demonstrated clinical effectiveness across a spectrum of ocular surface conditions, encompassing dry eye disease, Sjögren's syndrome, graft-versus-host disease, and neuropathic corneal pain (NCP). Currently, management of NCP remains challenging. The emergence of blood-derived eye drops represents a promising therapeutic approach. In this review, we discuss the benefits and limitations of different blood-derived eye drops, their mechanisms of action, and treatment efficacy in patients with NCP. Several studies have demonstrated the clinical efficacy of autologous serum eye drops in relieving pain and pain-like symptoms, such as allodynia and photoallodynia. Corneal nerve parameters were also significantly improved, as evidenced by increased nerve fiber density, length, nerve reflectivity, and tortuosity, as well as a decreased occurrence of beading and neuromas after the treatment. The extent of nerve regeneration correlated with improvement in patient-reported photoallodynia. Cord plasma eye drops also show potential for symptom alleviation and corneal nerve regeneration. Future directions for clinical practice and research involve standardizing preparation protocols, establishing treatment guidelines, elucidating underlying mechanisms, conducting long-term clinical trials, and implementing cost-effective measures such as scaling up manufacturing. With ongoing advancements, blood-derived eye drops hold promise as a valuable therapeutic option for patients suffering from NCP.

## Introduction

The concept of utilizing blood-derived products as therapeutic eye drops for management of ocular surface diseases was introduced in 1975. A pioneering effort was made by developing an ocular perfusion pump designed to administer autologous serum or plasma directly to the ocular surface of individuals suffering from chemical burns.^1^ Building upon this innovation, a significant milestone was reached a decade later when the application of autologous serum eye drops (SEDs) gained recognition as a potential treatment for dry eye associated with Sjögren's syndrome.^2^

Subsequently, this breakthrough paved the way for the introduction of various blood-derived products within the field of ophthalmology. These products are prepared either from the patient's peripheral blood serum (autologous source), such as autologous serum, platelet-rich plasma (PRP), plasma rich in growth factors (PRGF), and platelet lysate, or from donors, such as allogenic serum and umbilical cord serum (UCS).^3^ Nowadays, they have been clinically employed in a variety of ocular surface diseases, including moderate to severe dry eye syndrome,^3,4^ Sjögren's syndrome,^5^ graft-versus-host disease (GVHD),^6^ persistent epithelial defects (PED),^7^ neuropathic corneal pain (NCP),^8^ neurotrophic keratitis (NK),^9^ and even ocular surface disease caused by glaucoma treatments.^10^

## Different Types of Blood-Derived Eye Drops

### Autologous SEDs

Autologous SEDs are prepared from patients' peripheral blood serum under sterile conditions. They have been used as an effective treatment for dry eyes,^11,12^ NK,^13^ recurrent erosion syndrome,^14^ PED,^7,15^ and superior limbic keratoconjunctivitis.^16^ The rationale behind their efficacy lies in their composition, which closely resembles that of natural tears. Autologous SEDs contain an array of growth factors and cytokines, such as epithelial growth factor (EGF), fibroblast growth factor (FGF), vascular endothelial growth factor (VEGF), nerve growth factor (NGF), transforming growth factors (TGF-α and TGF-β1/β2/β3), insulin-like growth factor (IGF)-1, and IGF-2, as well as albumin, transferrin, α-2 macroglobulin, fibronectin, and vitamins.

Research has indicated that epitheliotrophic factors within autologous SEDs remain stable for up to 6 months when properly stored at −20°C.^17^ This complex mixture provides essential nutritional elements crucial for maintaining cellular tropism and epithelial migration, reducing the risk of infection, and suppressing apoptotic reactions during epithelial repair processes.^18^ Moreover, autologous SEDs harbor immunoglobulins (Ig) such as IgG, IgA, and lysozyme, imparting bactericidal and bacteriostatic effects.^18^ Through these biochemical factors, autologous SEDs have been shown to improve tear production, tear break-up time, goblet cells, and mucin expression in the conjunctiva.^19^

Despite their efficacy in treating various ocular disorders, autologous SEDs are accompanied by several drawbacks. Logistically, collecting blood and processing it into autologous SEDs may result in a considerable waiting time.^20^ Despite being prepared under sterile conditions, there remains a risk of contamination and potential infection during preparation, storage, and application of drops.^21,22^ Moreover, the largest barrier at this time is probably economic cost. In the United States, treatment ranges from $175 to $250 for a 2-month supply.^4^

Although there are numerous encouraging findings, the efficacy of autologous SEDs has been called into question by recent studies. A comparative study involving 34 patients did not observe the effectiveness of autologous SEDs in secondary Sjögren's syndrome, possibly attributed to elevated levels of proinflammatory cytokines in the serum.^23^ Furthermore, a prospective study involving 17 patients with dry eye syndrome illustrated the temporary advantages of autologous SEDs, lasting only up to 3 months after conclusion of the therapy.^24^

Additionally, a few contraindications to autologous SEDs exist, including patient-specific conditions such as poor venous access, low hemoglobin level, fear of needles, infants, and patients who are ineligible to make a blood donation due to comorbidities such as previous cardiovascular diseases. In old age, autologous SEDs might also pose challenges due to potential difficulties in blood collection.^20^ In these circumstances, allogenic blood-derived products may be more feasible.

### Allogenic SEDs

Allogenic SEDs are derived from blood serum obtained from healthy donors. They comprise identical components as autologous SEDs, but originate from different sources, making them equally effective for a range of ocular surface diseases, including dry eyes, PED, keratoconjunctivitis sicca, and chronic GVHD.^25–27^ Notably, a study reported the equivalence between allogenic SEDs and autologous SEDs in management of patients with severe dry eye disease, showcasing similar improvement in the ocular surface disease index (OSDI) and tear break-up time.^20^

However, the use of allogenic SEDs also comes with its risks, including transmission of blood-borne pathogens, hypersensitivity, and immune reactions.^28,29^ Hence, it is strongly recommended that the donor be tested for blood-transmitted diseases. In adherence to the guidelines established by the Bundesärztekammer and the Paul Ehrlich Institute concerning blood donation and utilization of blood products, screening involves testing for hepatitis B (HBV) and C (HCV), syphilis, and HIV serology (including HBsAg and antibodies to HCV, HIV-I/HIV-II, HIV NAT, syphilis, and HCV NAT).^21^

Additionally, in accordance with the National Blood Service (NBS) in England and Wales, routine virology testing aligns with the standards for volunteer blood donations in the United Kingdom. This testing encompasses screening for HBsAg and antibodies to HCV, HIV I/II, human T cell lymphoma virus (HTLV), and syphilis, along with HCV NAT.^21^

### Umbilical cord serum

UCS is derived from mothers during delivery. To prevent the spread of blood-borne infections, screening for infectious diseases must be conducted, following the same guidelines as described for allogenic eye drops.^21^ UCS consists of antibacterial agents (IgG and lysozyme) that provide bacteriostatic effects.^30^ It is known to contain higher amounts of growth factors, such as EGF, TGF, NGF, and VEGF, than other blood-derived preparations.^31^

EGF is a potent mitogen for epithelial cells,^32^ with varying effects on cell proliferation and number. Topical application of EGF at concentrations of 10 to 20 μg/mL has been reported to enhance corneal wound healing.^32^ TGF induces proliferation and migration of corneal stromal fibroblasts, affecting extracellular matrix synthesis and influencing the response to growth factors after injury.^33^ Recent studies propose that VEGFs may contribute to the natural repair of corneal nerves and play a crucial role in recovering from nerve damage in keratopathy.^34^

Moreover, UCS has higher levels of vitamin A and IGF-1 than peripheral blood serum.^32^ Vitamin A is essential for maintaining and repairing the ocular surface and supporting cell migration, growth, and differentiation. Furthermore, IGF-1 promotes the growth of keratinocytes, enhances the synthesis of N-cadherin, stimulates extracellular matrix formation, and increases collagen synthesis in keratinocytes.^35^

Similar to SEDs, UCS demonstrates versatile applications in various ocular pathologies, such as dry eye disease, GVHD, PED, recurrent corneal erosions, chemical burns, and NK.^36–41^ In cases of GVHD, UCS exhibits significant improvement in corneal sensitivity, tear break-up time, and corneal staining scores.^38^ Patients with NK experience improvements in corneal sensitivity and healing time with the UCS intervention.^41^ Of note, in a randomized controlled trial, UCS was found to be superior to autologous SEDs in terms of the corneal reepithelialization rate in PED.^37^

In a study involving patients diagnosed with severe dry eye disease and PED, participants were randomly assigned to either group A (treated with cord blood serum) or group B (treated with phosphate-buffered saline).^42^ Both groups received eye drops 8 times a day for 1 month. Group A had a more significant reduction in corneal staining. While both groups showed reductions in the visual analog score (VAS) and OSDI scores, group A reported significantly less grittiness and pain.^42^

Another prospective study explored morphological changes in corneal epithelium and sub-basal nerves using *in vivo* confocal microscopy (IVCM) in individuals with ocular surface disease undergoing cord blood SED treatment.^43^ Following the treatment, notable reductions were observed in OSDI, VAS, and Oxford grading values. Additionally, corneal sensitivity, Schirmer test score, and break-up time demonstrated significant increases. Positive alterations in corneal nerve morphology were noted, including an increased total nerve count and decreased tortuosity. Furthermore, there was a decrease in the presence of giant epithelial cells, beading, and neuromas.^43^

UCS can be used in patients with contraindications to autologous SEDs, as explained for allogenic SEDs. Moreover, a larger amount of serum or plasma can be collected from the umbilical vein at one time, which can improve the logistics of treatment and enable patients to experience therapeutic benefits without the need for prolonged waiting periods or additional preparation steps.^30^

### Platelet-derived eye drops

Platelets, integral components of blood-derived materials, encapsulate mediators within their α-granules that facilitate wound healing and tissue repair processes. These mediators encompass platelet-derived growth factor (PDGF), TGF-β1, IGF-1, EGF, VEGF, FGF, and hepatocyte growth factor (HGF).^44^ Among these, EGF and fibronectin play crucial roles in the proliferation and migration of epithelial cells, while PDGF, HGF, and FGF stimulate cell proliferation.^45^ Different preparations, namely PRP, PRGF, and platelet lysate, have been used for treatment of dry eye, PED, postrefractive dry eye, chemical injuries, and GVHD.^46–48^

The therapeutic impacts of PRP primarily stem from PDGF, which initiates wound healing by bolstering cell repair and stimulating angiogenesis.^49^ In an alternative preparation, platelet lysate can be produced by freeze–thawing platelets derived from PRP. This process facilitates the release of growth factors from platelets, driven by the action of thrombin.^50^ In contrast, PRGF, lacking thrombin,^51,52^ is produced by subjecting PRP derived from peripheral blood to centrifugation. The resulting PRP is then mixed with saline, frozen, and thawed to induce platelet lysis and subsequent release of PDGFs.

Several studies have made a comparative analysis of concentrations of the epitheliotrophic factor in autologous SEDs and various types of platelet-derived eye drops. These investigations reveal that platelet-derived eye drops exhibit higher levels of epitheliotrophic factors compared with autologous SEDs.^53,54^ Kim et al. reported a higher rate of corneal epithelium healing in cases of PED treated with PRP compared with autologous SEDs.^54^ Another group highlighted that PRP could potentially be more effective than autologous serum as a greater extent of improvement in the ocular surface assessment, such as Schirmer's value, was seen in the PRP group compared with the autologous SED group.^55^ Furthermore, PRP exhibited the advantage of requiring a shorter preparation time.^55^

Nevertheless, variations in preparation methodologies may influence data interpretation.^55,56^ Addressing concerns regarding the stability of these epitheliotrophic factors during storage is another critical consideration. A study focusing on PRGF eye drops highlights that certain components such as PDGF-AB, VEGF, EGF, and vitamin A remain stable when stored at −20°C for up to 90 days, whereas others such as fibronectin and TGF-β1 did not show stability under the same conditions.^56^ These emphasize the importance of optimizing preparation techniques and storage conditions to harness the therapeutic potential of these eye drops effectively.

In a 6-week prospective study, 44 eyes from 28 patients with corneal ulcers after corneal surgery received autologous PRP. The results revealed a reduction in ulcer size for 26 patients (59.1%), and an overall improvement in symptoms, such as relief from foreign body sensation and pain, was observed in 40 patients (90.9%).^57^

Wang et al. assessed the safety and effectiveness of combined PRGF eye drops and scleral contact lens therapy for ocular surface disease.^58^ The study found that the use of concurrent therapy significantly decreased the severity and frequency of dry eye symptoms. Patient satisfaction with the combined therapy was high, with a majority of patients considering it better than previous treatments.^58^

### Cord blood platelet lysate

Cord blood has recently emerged as a promising alternative for platelet lysate production, offering cost-effectiveness and reduced batch-to-batch variability.^59^ Cord blood platelet lysate contains higher protein levels and elevated concentrations of growth factors such as PDGF-AB, TGF, EGF, FGF, and HGF when compared with peripheral blood platelet lysate. These growth factors remain stable even after 9 months of storage at −70°C.^59^ Table 1 summarizes the characteristics of different types of blood-derived eye drops.

**Table 1.** Comparisons of Different Types of Blood-Derived Eye Drops

Blood-derived eye drops	Source	Benefits over other preparations
Autologous serum	Patient's own blood	Lower risk of disease transmission.
Allogenic serum	Healthy donor	Can be used in case of contraindications to autologous source. Infants Elderly Comorbidities such as previous cerebrovascular accidents or cardiovascular disease or anemia
Umbilical cord serum	Umbilical cord from a donor	Can be used in case of contraindications to autologous source. Infants Elderly Comorbidities such as previous cerebrovascular accidents or cardiovascular disease or anemia Better logistics of treatment Less prolonged waiting periods
Platelet-derived serum	Patient's blood	Higher levels of epitheliotrophic factors
Cord blood platelet lysate	Umbilical cord from a donor	Can be used in case of contraindications to autologous source. Infants Elderly Comorbidities such as previous cerebrovascular accidents or cardiovascular disease or anemia Contains higher concentrations of growth factors compared with peripheral blood platelet lysate

## Neuropathic Corneal Pain

NCP is a debilitating condition in which the nociceptive system and somatosensory pathway are disrupted, leading to hyperactive pain signaling and disproportionate pain with increased sensitivity to external stimuli.^60^

It can result from various etiologies, encompassing ocular causes, including ocular surface disorders such as chronic dry eye disease^61^; recurring corneal erosions^62^; corneal infections such as herpes simplex and herpes zoster^63^; trauma^62^; corneal, ocular, or refractive surgeries^64^; and radiation-induced keratopathy,^62^ as well as systemic causes, including conditions such as diabetes,^65,66^ fibromyalgia,^62^ small-fiber polyneuropathy,^62^ chemotherapy, autoimmune conditions (inflammatory bowel disease, celiac diseases, sarcoidosis, lupus, and Sjögren's syndrome), and trigeminal neuralgia.^67^

Moreover, anxiety and depression are the comorbidities that are associated with NCP^62^ (Table 2). NCP results in discordance between symptoms and clinical signs, as patients may exhibit a completely normal or near-normal-looking ocular surface on slit lamp evaluation despite experiencing significant discomfort.^68^

**Table 2.** Etiologies of Neuropathic Corneal Pain

Ocular causes	Systemic causes
Ocular surface disorders such as chronic dry eye disease^61^ and recurrent corneal erosions^62^ Corneal infections such as herpes simplex and herpes zoster^63^; trauma^62^; corneal, refractive, or ocular surgeries^64^; and radiation-induced keratopathy^62^	Diabetes^65,66^ Fibromyalgia^62^ Small-fiber polyneuropathy^62^ Chemotherapy^67^ Autoimmune conditions (inflammatory bowel disease, celiac disease, sarcoidosis, lupus, and Sjögren's syndrome)^67^ Trigeminal neuralgia^67^ Comorbidities: anxiety and depression^62^

### Diagnosis of NCP

Diagnosis of NCP is challenging, and no consensus has been reached. Currently, NCP diagnosis relies on symptomatic presentation, such as pain, allodynia, photoallodynia, or burning sensation,^69,70^ as well as clinical examinations. The ocular surface presents with intact integrity or minimal staining. A recent study showed that patients with NCP present with increased corneal sensitivity,^71^ as opposed to another study that reported a decrease in sensitivity.^72^ Additionally, assessment of nerve status is facilitated by imaging modalities such as IVCM, which enable visualization of corneal nerve fibers.^73^

**FIG. 1.**
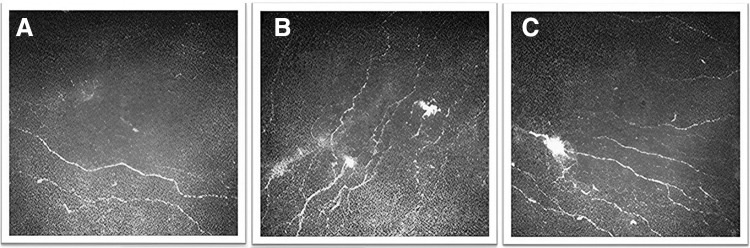
Representative IVCM images of the corneal sub-basal nerve plexus and the presence of microneuroma in NCP patients. **(A)** Sub-basal nerves with decreased density. **(B)** The presence of spindle microneuromas, which appear as hyper-reflective fusiform enlargement of the nerves. **(C)** The presence of stump microneuroma, which is characterized by terminated swelling of the nerve. IVCM, *in vivo* confocal microscopy; NCP, neuropathic corneal pain.

Patients with NCP have demonstrated decreased corneal nerve density on IVCM.^74,75^ They also presented with microneuromas, indicative of abrupt nerve terminal ending enlargement following injury, which is distinct from NCP,^76,77^ thereby suggesting a potential diagnostic significance (Fig. 1). While microneuromas can be observed in individuals with NCP, they can also be detected in both individuals experiencing symptoms of dry eyes and those without such symptoms.^78^

A recently validated tool in the field of ocular pain assessment is the Ocular Pain Assessment Survey (OPAS),^67^ which is a quantitative and multidimensional questionnaire to evaluate corneal and ocular surface pain, along with its associated quality of life implications. This comprehensive assessment framework permits a quantitative evaluation of treatment responses, enabling practitioners to monitor the effectiveness of interventions.^67^ The proparacaine challenge test is used to distinguish between central and peripheral origins of NCP, which is crucial when determining treatment options.^79^

### Management of NCP

Management strategies for NCP require differentiation of peripheral, mixed, and centralized sources of pain. A multidisciplinary approach is often recommended, involving collaboration between ophthalmologists, neurologists, pain specialists, rheumatologists, psychiatrists, and other health care professionals.

### Ocular surface treatment

Lubricants aid in dilution of inflammatory mediators and decrease tear film hyperosmolality. They also provide better spread of the lipid layer of tears.^79^ Punctal plugs increase retention of tears.^80^ In cases of blepharitis, hot compresses and lid massage can be done.^79^

### Anti-inflammatory agents

Topical steroids are the mainstay of treatment through their inhibitory action on cytokines, prostaglandins, and leukotriene synthesis, as well as inhibition of leukocyte migration.^81,82^ Due to ocular complications associated with prolonged topical corticosteroid use, steroid-sparing agents such as topical 0.05% cyclosporine A^83^ or 0.03% tacrolimus^84^ can be considered. Cyclosporine functions by inhibiting T cell activation and suppressing inflammatory cytokines such as IL-2, IL-6, IL-12, tumor necrosis factor alpha, interferon gamma, and granulocyte–macrophage colony-stimulating factor.^83^

Tacrolimus shares a mechanism of action similar to cyclosporine A; however, it is reported to be 10 to 100 times more potent.^84^ The emergence of 5% lifitegrast, a novel anti-inflammatory class, has also broadened therapeutic options.^85^

### Amniotic membrane transplantation and protective contact lenses

The cryopreserved amniotic membrane has demonstrated multifaceted benefits for the ocular surface, including its anti-inflammatory, antifibrotic, and neurotrophic effects.^86^ Application of the amniotic membrane demonstrates promising outcomes in individuals with NCP, quickly alleviating symptoms, particularly pain.^67^ Patients with peripheral NCP unresponsive to topical treatments may find relief through temporary trials with extended-wear soft bandage contact lenses (CLs) or scleral lenses.

For immediate symptom alleviation, a scleral device known as prosthetic replacement of the ocular surface ecosystem has been employed, leading to reduced pain as reported by patients.^87,88^ These methods likely shield corneal nociceptors from external environmental triggers. Scleral lenses have shown effectiveness in reducing light sensitivity and discomfort.^89^ Nonetheless, the use of CLs can pose challenges for those with severe underlying hyperalgesia, as the lenses may induce noxious stimuli in such cases.

### Systemic pharmacotherapy

When central neuropathic pain components are involved, the treatment of NCP often relies on systemic pharmacotherapy.^62,90^ Randomized studies addressing their use in NCP are lacking and their application can be inferred from their successful use in treating neuropathic pain elsewhere.^91^

Drugs such as gabapentin and pregabalin, initially developed as anticonvulsants, bind to α2-δ subunit voltage-gated calcium channels, preventing the release of glutamate, norepinephrine, and substance P. This action stabilizes neurons, making them effective primary agents for addressing neuropathic pain associated with conditions such as diabetic neuropathy, postherpetic neuralgia, and central neuropathic pain.^92^ Tricyclic antidepressants inhibit presynaptic reuptake of serotonin and norepinephrine and block cholinergic, histaminergic, and sodium channels. The International Association for the Study of Pain suggests prioritizing the use of secondary amine tricyclic antidepressants, such as nortriptyline and desipramine, as the preferred initial treatment for neuropathic pain.^93^

Serotonin–norepinephrine inhibitors have also been explored for neuropathic pain treatment.^92,93^ Carbamazepine, an anticonvulsant acting as a sodium channel blocker, is commonly used for trigeminal neuralgia.^94^ It alleviates corneal pain at low doses, while opioids such as tramadol bind to μ-opioid receptors and are effective in moderate to severe pain.^92,93^ Low-dose naltrexone, an opioid antagonist, also exhibits antagonist effects on Toll-like receptor 4, linked to neuropathic pain, reducing proinflammatory cytokine release.^95^

Duloxetine and venlafaxine, serotonin–norepinephrine reuptake inhibitors, possess both antidepressant and central analgesic properties, with duloxetine FDA approved for painful diabetic polyneuropathy.^96^ Mexiletine, a sodium channel blocker, is an orally active local anesthetic agent. It is structurally related to lidocaine and serves as a second- or third-line treatment for neuropathic pain.^97^

### Complementary and alternative therapies

Adjunctive therapies such as acupuncture and cardio exercise have demonstrated temporary relief and potential reduction in the need for pharmacotherapy.^98^ Neuromodulation, involving electrical or chemical agents, modifies neural activity for therapeutic outcomes.^99^ Dietary interventions, such as omega-3-to-omega-6 ratio optimization, offer additional avenues for managing NCP.^100^

### Blood-derived preparations

Recently, blood-derived eye drops have gained attention as a potential therapeutic approach for NCP. A growing body of literature has emerged focusing on the potential role.

## Blood-Derived Eye Drops for Treatment of NCP

In a retrospective case–control study, a significant reduction in corneal pain scores among individuals with NCP was observed following the application of 20% SEDs for ∼3.8 months. Comparing 16 patients suffering from severe NCP with 12 control subjects without ocular surface diseases, the study documented pain severity and various corneal nerve characteristics. Sub-basal nerves were significantly decreased before treatment in the NCP group compared with controls. Following treatment with SEDs, the average pain severity (0–10) significantly decreased from 9.1 ± 0.2 to 3.1 ± 0.3.

IVCM further revealed significant improvement in total nerve length and number, coupled with significant reductions in reflectivity and tortuosity. The underlying hypothesis posits that autologous SEDs, enriched with a spectrum of neurotrophic and growth factors, play a pivotal role in nerve regeneration, facilitating the repair of aberrant nerves, restoring function in damaged neurons, and therefore ameliorating pain.^8^

In 2 additional retrospective case–control studies, the potential efficacy of applying 20% autologous SEDs 8 times daily over 3.6 months^69^ and 3.8 months^101^ was investigated as a therapeutic intervention for individuals suffering from persistent, debilitating corneal pain associated with corneal neuropathy. The severity of photoallodynia symptoms was recorded based on the patient's assessment on a scale of 0–10, 10 being the maximum.

The outcomes showed a significant improvement in allodynia symptoms,^69^ as well as a correlation between nerve regeneration and improved patient-reported photoallodynia.^69^ After autologous SED treatment, patients reported remarkable symptom reduction, with 56.3% (9/16) experiencing over 90% improvement and the remaining 43.8% (7/16) reporting 40%–60% improvement in their photoallodynia symptoms. The average symptom severity significantly decreased from 8.8 ± 1.1 to 1.6 ± 1.7.

Furthermore, IVCM findings demonstrated significantly improved nerve parameters, including total nerve length, nerve number, and nerve reflectivity. Following treatment, the occurrence of beading and neuromas significantly reduced to 56.2% and 7.6%, respectively, marking a 37.5% decrease in beading and 56.3% decrease in neuromas when compared with their pretreatment levels. When clinical symptom severity was correlated with IVCM parameters, a significant negative correlation was found between photoallodynia symptom severity and total nerve density, while a significant positive correlation was observed between symptom severity and reflectivity of nerves.^69^

In another study, following a treatment duration of 3.8 ± 0.5 months with autologous SEDs,^101^ there was a significant improvement in the severity of corneal pain, reducing from a baseline score of 9.4 ± 0.2 to 3.4 ± 0.4. Baseline IVCM analysis revealed significantly reduced density and altered morphology of the sub-basal nerve plexus in all measured parameters compared with controls.

In alignment with symptomatic improvement, a significant improvement in the nerve plexus was observed postautologous SED treatment. This improvement was reflected in the total nerve count and density and branch nerve count and density, as well as reduced nerve reflectivity and tortuosity. Morphologically, in all patients, the presence of nerve beading and neuromas significantly decreased from 37.5% and 75% to 6.3% and 68.8%, respectively.^101^

These studies underscored the role of autologous SEDs, enriched with diverse neurotrophic factors, in driving the regeneration of corneal sub-basal nerves, ultimately leading to significant corneal pain improvement. Notably, IVCM indicated a significant increase in corneal sub-basal nerve number and density, alongside a decrease in nerve beading, reflectivity, tortuosity, and neuroma presence.^69,101^

**FIG. 2.**
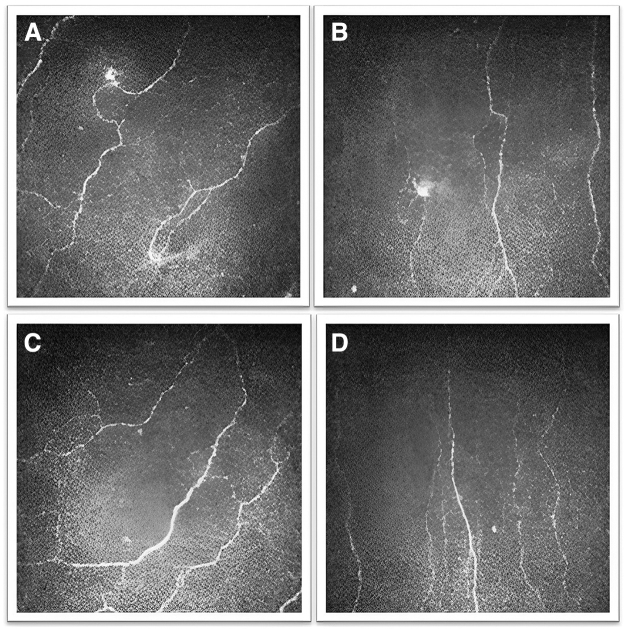
Representative IVCM images of the changes of the sub-basal nerve plexus in an NCP patient before and after cord plasma eye drop treatment. **(A, B)** Decreased corneal nerve density and the presence of microneuroma were observed before treatment. **(C, D)** An increase in corneal nerve density and a marked decrease in the microneuroma were observed after treatment.

Cord plasma eye drops can also be a potential therapeutic agent for NCP. Our group observed that the size and perimeter of corneal neuromas significantly decreased, the corneal nerve density and length were significantly restored, and patients' subjective pain or pain-like symptoms, particularly photophobia, significantly ameliorated after 6 months of topical cord plasma treatment (Fig. 2; unpublished data).

In a study comparing deproteinized calf blood extract (DCBE) and 0.3% sodium hyaluronate (SH), the group treated with DCBE demonstrated superior improvement in OSDI light sensitivity and ocular pain scores (*P* < 0.05).^102^ Both DCBE and SH groups showed significant enhancement in tear break-up time, Schirmer test score, corneal fluorescein staining, OSDI score, light sensitivity score, and ocular pain score at 2 and 4 weeks post-treatment (*P* < 0.05) compared with pretreatment data. This study suggests that DCBE eye drops are more effective in alleviating ocular pain and light sensitivity in dry eye patients compared with SH eye drops.^102^

Several studies have delved into the intricate mechanisms underlying the therapeutic effects of blood-derived components on pain relief in NCP. Blood-derived eye drops that are rich in NGF are effective in the treatment of nonocular neuropathic pain in a rat model. Significant amelioration in allodynia and thermal hyperalgesia was observed following intrathecal infusion of NGF in rats with spinal cord injury, suggesting a potential avenue for managing pain with neurotrophic factors.^103^ The results of clinical studies reporting the efficacy of blood-derived eye drops are summarized in Table 3.

**Table 3.** Summary of Studies Investigating the Effects of Blood-Derived Eye Drops in Neuropathic Corneal Pain

Study	Study design	*N *(number of patients)	Key findings
Aggarwal et al.^8^	Case–control study	Cases: 16 patients with NCP Controls: 12 subjects	Autologous SEDs were beneficial for significant alleviation of corneal pain observed after 3.8 months of use and improvement in nerve metrics, including total nerve length, nerve number, decrease in nerve reflectivity, and decrease in nerve tortuosity
Aggarwal et al.^69^	Case–control study	Cases: 16 patients who have photoallodynia due to corneal neuropathy Controls:16 subjects	Autologous SEDs provide benefits of significant improvement in allodynia symptoms; decrease in sub-basal corneal nerve beading and presence of neuromas; significant improvement in nerve parameters, including total nerve length, nerve number, and nerve reflectivity; and nerve regeneration that correlated with improvement in patient-reported photoallodynia
Aggarwal et al.^101^	Case–control study	Cases: 16 patients who have severe corneal pain due to corneal neuropathy Controls: age-matched controls = 12	Autologous SEDs showed significant improvement in the severity of corneal pain and improvement in corneal nerve number and density along with a decrease in nerve beading, reflectivity, tortuosity, and presence of neuromas
Wu et al.^102^	Randomized controlled trial	Total: 53 deproteinized calf blood extract group (*n* = 22) and 0.3% sodium hyaluronate group (*n* = 31)	Deproteinized calf blood extract eye drops relieved ocular pain and light sensitivity in dry eye patients better than SH eye drops.

SEDs, serum eye drops.

In addition to working through neurotrophic factors, several mechanisms may also explain the treatment effects of blood-derived eye drops on NCP. Exosomes, which are naturally occurring components found in bodily fluids, including serum, act as carriers for an array of regenerative factors such as growth factors, lipids, and microRNAs.^104^ MicroRNAs play crucial roles in the development and advancement of neuropathic pain by modulating neuronal excitability and plasticity, as well as influencing neuronal ion channels, neuroinflammation, synaptic plasticity, and various other functions.^105^

Additionally, lipids, especially specialized proresolving lipid mediators, possess anti-inflammatory properties that aid in resolving inflammation.^106^ Neurons subjected to exosome treatment have displayed enhanced neurite growth and increased complexity.^107^ Furthermore, corneal transient receptor potential vanilloid 1 (TRPV1) is critical to the function of polymodal nociceptors.^108^ TRPV1+ nerves have been shown to account for a higher proportion of corneal nerves after injury, which may provide insights into the pathophysiology of neuropathic pain.^109^

TRPV1 functions as a nonselective cation channel, responding to stimuli such as heat, protons, and vanilloid compounds such as capsaicin.^108^ It is generally associated with pain sensation and has been reported to mediate corneal nociception.^110^ Substance P (SP) at the physiological level is important to ocular surface integrity; however, excessive SP at the ocular surface is detrimental, inducing inflammation and causing pain.^110–112^ Activation of TRPV1 triggers the release of neuropeptides such as calcitonin gene-related peptide and neurokinins or SP, leading to the experience of pain and inflammation.^113^

Inhibitors specifically targeting the TRPV1 channel have demonstrated efficacy in diminishing inflammatory, traumatic, nociceptive, and neuropathic pain in both human and animal studies.^114–116^ This inhibition helps mitigate the proinflammatory consequences associated with TRPV1 signaling. Notably, TRPV1 antagonists exhibit potent topical analgesic effects without induction of topical anesthesia.^113^ This characteristic renders them valuable for alleviating symptoms of ocular pain.^113^

Ferrari et al. highlighted the significant involvement of SP in regulating ocular surface pain as it enhances the sensitivity of pain receptors, amplifies pain signals, and promotes inflammation in the nervous system. Targeting its receptor, neurokinin 1, presents a promising therapeutic strategy for effectively managing ocular pain by decreasing SP release and limiting leukocyte infiltration within the cornea.^117^ Moreover, tear hyperosmolarity, in conjunction with tear instability, stands as the primary trigger that initiates damage to the ocular surface, ultimately resulting in pain.^118^

**FIG. 3.**
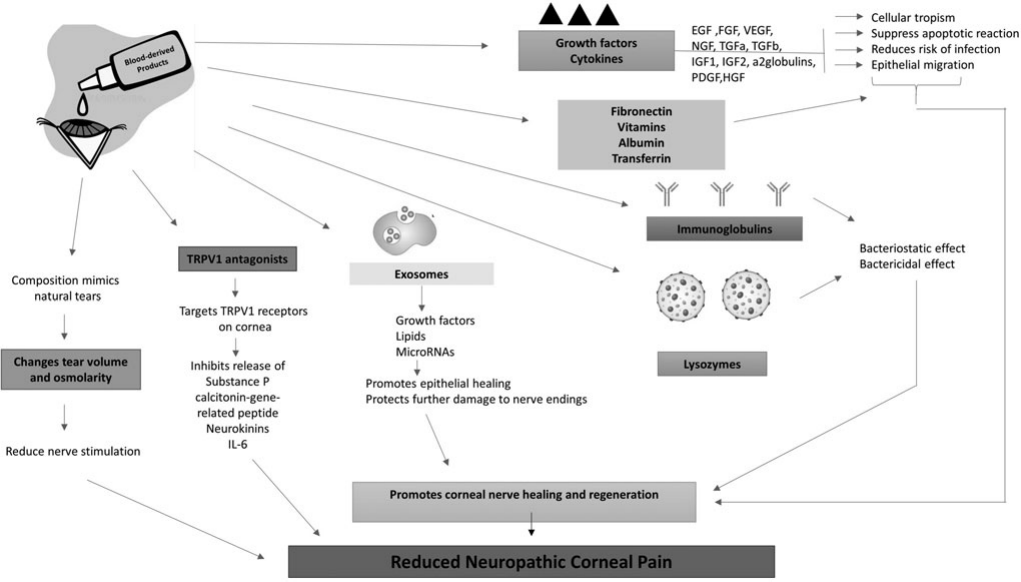
Postulated effects of blood-derived eye drops on NCP.

Blood-derived preparations, characterized by compositions that closely mimic natural tears,^119^ alter the tear volume or osmolarity, thereby preventing further damage to the ocular surface, reducing nerve stimulation, and ultimately alleviating corneal pain. The postulated effects of blood-derived eye drops on NCP are illustrated in Fig. 3.

## Future Directions

As the field of blood-derived eye drops for NCP continues to evolve, several avenues for future clinical development and research emerge. First, the optimal concentration, as well as frequency, has to be ascertained. Second, there are inconsistencies in the preparation of these blood-derived eye drops, which have to be standardized. Establishing treatment guidelines would enable consistent and evidence-based treatment approaches across different health care settings.

Third, further elucidation of the specific mechanisms by which blood-derived components alleviate pain is crucial. These require in-depth molecular studies to uncover the interplay between neurotrophic factors, biological processes, corneal nerve imaging characteristics, and clinical symptoms involved in neurobiological repair.

Monitoring for potential side effects (such as infections, allergic reactions, redness, or discomfort upon application) and assessing the impact of the treatment on overall ocular surface health will be important. Long-term clinical trials focusing on larger scale cohorts will provide more comprehensive insights into the long-term effectiveness. Future endeavors to decrease the cost of blood-derived SEDs involve scaling up and innovative approaches.

Research into optimized blood plasma collection and processing techniques holds the potential to improve cost efficiency. Progress in biomanufacturing methods may boost effectiveness and decrease labor costs. Additionally, ongoing evaluation of production, packaging, and distribution approaches will establish a sustainable trajectory for enhancing affordability and availability.

## Conclusions

The exploration of blood-derived eye drops as a therapeutic strategy for NCP represents a significant stride in NCP management. The studies discussed in this review collectively highlight the potential of blood-derived eye drops, containing growth factors, cytokines, and other bioactive components, to promote corneal nerve regeneration as well as repair nerve injury, thereby relieving pain.

With continued research, clinical validation, cost-effective strategies, and technological progress, blood-derived eye drops have the potential to become a mainstream therapeutic option for individuals suffering from this debilitating condition.
